# Intervertebral disc kinematics in active duty Marines with and without lumbar spine pathology

**DOI:** 10.1002/jsp2.1057

**Published:** 2019-06-17

**Authors:** Keenan Onodera, David B. Berry, Bahar Shahidi, Karen R. Kelly, Samuel R. Ward

**Affiliations:** ^1^ Department of Orthopaedic Surgery University of California San Diego La Jolla California; ^2^ Department of Bioengineering University of California San Diego La Jolla California; ^3^ Department of Radiology University of California San Diego La Jolla California; ^4^ Warfighter Performance Department Naval Health Research Center San Diego California

**Keywords:** intervertebral disc bulge, intervertebral disc degeneration, low back pain, lumbar spine, upright MRI

## Abstract

Military members are required to carry heavy loads frequently during training and active duty combat. We investigated if operationally relevant axial loads affect lumbar disc kinematics in forty‐one male active duty Marines with no previous clinically diagnosed pathology. Marines were imaged standing upright with and without load. From T2‐weighted magnetic resonance images, intervertebral disc (IVD) health and kinematic changes between loading conditions and across lumbar levels were evaluated using two‐way repeated measures analysis of variance tests. IVD kinematics with loading were compared between individuals with and without signs of degeneration on imaging. Linear regression analyses were performed to determine associations between IVD position and kinematic changes with loading. Fifty‐eight percent (118/205) of IVDs showed evidence of degeneration and 3% (7/205) demonstrated a disc bulge. IVD degeneration was not related to posterior annular position (*P* > .205). Changes in sagittal intervertebral angle were not associated with changes in posterior annular position between baseline and loaded conditions at any lumbar level (*r* < 0.267; *P* = .091‐.746). Intervertebral angles were significantly larger in the lower regions of the spine (*P* < .001), indicating increased local lordosis when moving in the caudal direction Disc height at the L5/S1 level was significantly smaller (6.3 mm, mean difference = 1.20) than all other levels (*P* < .001) and baseline posterior disc heights tended to be larger at baseline (7.43 mm ± 1.46) than after loading (7.18 ± 1.57, *P* = .071). Individuals with a larger baseline posterior annular position demonstrated greater reduction with load at all levels (*P* < .002), with the largest reductions at L5/S1 level. Overall, while this population demonstrated some signs of disc degeneration, operationally relevant loading did not significantly affect disc kinematics.

## 1 INTRODUCTION

Military members are required to carry heavy loads frequently during training and combat. During operations, Marines carry a minimum operational load of 11.3 kg in the form of ballistic protection, which can quickly escalate with the addition of necessary equipment to over 45 kg, exceeding the recommended load carriage limit of 33 kg.^1^ Higher rates of intervertebral disc (IVD) degeneration has been observed to occur at a higher frequency in military populations compared to similarly‐aged civilians.^2^ It is thought that load‐induced changes in IVD health may play a role in the development of clinical back pathology in this population. However, the association between operational loading, disc degeneration, and clinical spinal pathology (ie, bulge, herniation) has not been explicitly explored.

Heavy axial loads alter natural spinal posture, which may fatigue paraspinal musculature necessary for stabilization.^3^ This may increase a Marines vulnerability to IVD injury and an increased rate of IVD degeneration over time.^4^ Furthermore, individuals with disc degeneration demonstrate not only decreased whole lumbar range of motion, but also decreased intervertebral range of motion, specifically at the levels with degenerated IVDs.^4^, ^5^, ^6^ This decreased range of motion may alter axial distribution of weight, affecting compression and shear forces at intervertebral joints.^7^, ^8^ Previous investigations on the effect of load and position on IVD kinematics (IVD height and intervertebral angular changes) in Marines demonstrated that as local lumbar flexion increased, decreased anterior and increased posterior IVD height occurs under operational loading conditions.^4^, ^9^ However, these previous investigations did not examine changes in posterior annular position (defined as focal or asymmetric extension of the disk beyond the vertebral border^10^) with load, or the influence of disc health on kinematic loading responses. Evidence of IVD kinematic changes in response to postural alterations suggests that axial loading may also affect more specific features of disc morphology, such as annular position.

Disc morphology is often used as an indicator of IVD health, and changes in disc morphology are observed with disc degeneration and injury.^11^ Changes in disc morphology with degeneration are thought to be a result of decreased proteoglycan concentration within the nucleus pulposus leading to loss of hydration and ultimately a decrease in disc height over time, or destabilization of the disc due to an annular or nucleus pulposus injury.^12^ Although IVD herniation is apparent and well defined, the current literature does not provide a clear clinical definition for the term *disc bulge,* implicating its dependence on individual patient characteristics. Furthermore, clinically relevant changes in kinematics could include, but are not limited to, significant posterior annular protrusion compressing neural elements, loss of IVD height mimicking fusion, and resultant intervertebral angular derangements.

In order to further understand the influences of load on IVD kinematics in active duty Marines, the purpose of our study was to (a) investigate the effect of operationally relevant load on IVD height, intervertebral angle, and posterior annular position in the lumbar spine, and (b) to compare IVD kinematics between Marines with and without disc degeneration. We hypothesized that under increased axial load from tactical equipment, Marines' lumbar IVDs would demonstrate increased posterior displacement of the annulus fibrosus compared to baseline. Additionally, we hypothesized that Marines with IVD degeneration would exhibit decreased disc height and IVD angles compared to those with nondegenerated IVDs.

## 2 METHODS

### 2.1 Study design

This is a retrospective analysis of lumbar spine imaging data with repeated‐measures design. Independent variables were loading condition and disc degeneration on intervertebral angle, posterior annular position, lordosis, and IVD height.

### 2.2 Volunteers

Utilizing patients from a previous study, a total of 43 male active duty Marines from the Marine Corps Base Camp Pendleton volunteered to participate. Participants were included if they were currently active‐duty Marines between the ages of 18‐45. Participants were excluded if they had undergone any spinal surgery, recent musculoskeletal injuries, or diagnosed spinal pathology, any metal implants or devices within their body, including but not limited to prosthetic devices, shrapnel, and surgical implants that could compromise the safety of participating in an magnetic resonance imaging (MRI) scan. Due to motion artifact, two datasets were excluded from the study. Therefore, 41 of the previously acquired 43 participant datasets were included. The University of California, San Diego and Naval Health Research Center Institutional Review Boards approved this study, and all volunteers provided oral and written consent to participate.

### 2.3 Load carriage

Marines were scanned naturally standing without load and standing with body armor (11.3 kg). The 11.3 kg body armor was used because it is minimum protective equipment that Marines are required to wear during military operations/training. The body armor was retrofitted to remove any metallic components to ensure compatibility with MRI. Marines were not provided instruction on how to assume each position, but were asked to hold each position steady for the duration of the entire MRI acquisition (approximately 3 minutes).

### 2.4 Imaging

Marines were scanned in their natural standing position and standing under axial load (11.3 kg) using an upright 0.6 T MRI scanner (Upright Multi‐Position MRI; Fonar Corporation, Melville, New York) and a planar coil. An elastic band was used to hold the coil against the volunteer's lumbar spine between the L1‐S1 levels while standing. The band was secured to hold the coil in place while not altering the volunteer's natural position. A three‐plane localizer (TR = 1254 ms, echo time (TE) = 100 ms, field of view (FoV) = 34 cm, matrix = 256 × 256, resolution 1.33 mm × 1.33 mm, THK = 9 mm, NEX = 1, time = 0:17) and sagittal T2‐weighted images (TR = 1974 ms, TE = 160 ms, FOV = 35 cm, matrix = 224 × 224, resolution 1.56 mm × 1.56 mm, slice thickness (THK) = 3 mm, gap = 0 mm, number of excitations (NEX) = 1, time = 2:12) were acquired.

### 2.5 Image analysis

Postural measurements (IVD height, IVD angle) were generated from upright MRI images in each load configuration using a previously validated algorithm^13^ using OsiriX.^14^ Briefly, markers were manually placed on the corners of each lumbar vertebra and on the pedicles of each lumbar vertebra on consecutive sagittal MRIs using OsiriX. The locations of each marker were imported into MATLAB (MathWorks, Natick, Massachusetts) and used to define an endplate‐based joint coordinate system, which was applied to the superior and inferior endplates of each vertebra, from which local positional measurements were made. Previously, this method has been shown to have an average absolute error of 0.77° ± 0.55° for sagittal angle measurements and 1.74 mm ± 1.11 mm for intervertebral height measurements.^13^

**Figure 1:**
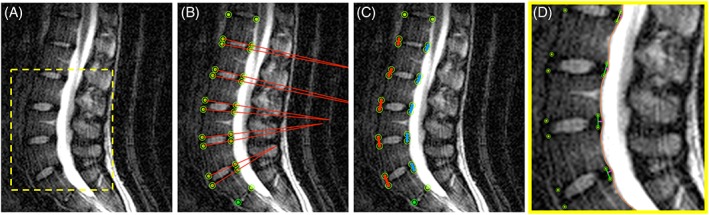
Schematic depicting measurement of A, Midsagittal lumbar spine. B, Intervertebral angle. C, Anterior (red) and posterior (blue) intervertebral height. D, Posterior annular position (pink)

Local measurements of lumbar spine posture including intervertebral angles and distances were obtained. *Sagittal intervertebral angle* was measured between the superior and inferior endplates of adjacent vertebrae to describe local changes in lordosis, and distribution of flexion/extension throughout the lumbar spine. Increases in IVD angle correspond to lumbar extension while decreases in IVD angle correspond to lumbar flexion. *IVD height* was measured as the anterior and posterior distances between contiguous vertebrae. Disc bulge was quantified as *posterior annular position*, defined as the maximum distance between a line connecting the posterior aspects of adjacent vertebrae and the posterior IVD border at the mid‐sagittal slice for each IVD (Figure 1). The mid‐sagittal slice was identified as the slice containing the spinous process for each intervertebral level.

We recorded posterior annular positions of the discs as a measure of disc bulge. Adapting previously established nomenclature, we defined *disc bulge* as a posterior annulus extending beyond the vertebral body^15^ greater than two SDs (>95th percentile) above the group mean posterior annular position. Those with posterior annular positions greater than the 95th percentile at baseline in the standing unloaded position were considered to have a disc bulge. Those with posterior annular positions less than the 95th percentile at baseline in the standing unloaded configuration were considered normal. Furthermore, all subjects were screened for paracentral disc bulge and IVD pathology from a high resolution, axial MRI acquired as part of a complimentary study.^16^

### 2.6 Disc grading

All lumbar discs were graded for disc degeneration using the Pfirrmann grading scale.^17^ The Marines' discs were separated into degenerated or nondegenerated groups based on Pfirrmann grade; IVDs with a Pfirrmann grade of III or more were assigned to the “degenerated” group and IVDs with a Pfirrmann grade of II or less were assigned to the “non‐degenerated” group. These classifications were made based on data supporting IVD biomechanical changes above grade II.^18^, ^19^, ^20^

### 2.7 Statistical analysis

Posterior annular position was analyzed using 2‐way repeated measures analysis of variance (ANOVA) tests (levels: load, IVD level) with post hoc Sidak tests to identify differences between load configurations and intervertebral levels. Secondary analyses were conducted to determine differences in local measurements of intervertebral angles and posterior disc heights under axial load and at baseline at all levels using the same statistical analyses. A separate 2‐way ANOVA (configuration × degeneration) with post hoc Sidak tests was performed to identify kinematic differences in discs with degeneration. Discs were grouped based on degeneration of the lumbar spine (degenerated, nondegenerated as defined above). Linear regression was used to determine the relationships between the posterior annular position of the IVDs at baseline and the change in posterior annular position with loading. Similarly, linear regression was used to determine relationships between the change in posterior annular position and change in intervertebral angles between baseline and loaded positions. As participant height is related to IVD height, which may affect the magnitude of disc bulge, a Pearson correlational analyses was used to explore if there was a relationship between volunteer height, IVD height, and posterior annular position. Due to the retrospective nature of this study, an a priori sample size estimation was not performed.

Data are expressed as mean ± SD, significance was set at *α* = .05, and *r* ^2^ values were used to express the strength of linear regression relationships. Statistics were computed using Prism 6 (GraphPad Software, Inc., La Jolla, California).

## 3 RESULTS

### 3.1 Participant demographics

Complete image datasets were analyzed from 41 male active duty Marines (Table 1). IVD degeneration assessed by the Pfirrmann grading scale (≥ grade III) was observed in 38/41 (93%) participants in at least one level and in 118/205 (58%) of all lumbar IVDs. No paracentral disc bulges, or IVD pathology was observed. No significant relationship between participant height and posterior annular position at any lumbar level was found (*P* > .05). Similarly, baseline disc height was not associated with changes in posterior annular position with load (*P* > .18).

**Table 1:** Demographics and IVD degeneration (Marines with degeneration in ≥1 lumbar level) for all volunteers

Study	No. of volunteers	Age (years)	Height (m)	Weight (kg)	BMI (kg/m^2^)	Degenerated IVDs (Pfirrmann ≥III)	Degeneration at ≥1 lumbar level
1^9^	n = 41	26.8 ± 6.4	1.78 ± 0.07	81.9 ± 9.8	25.9 ± 2.9	118/205	38/41

Abbreviations: BMI, body mass index; IVD, intervertebral disc.

### 3.2 Effect of IVD degeneration on posterior annular position

**Figure 2:**
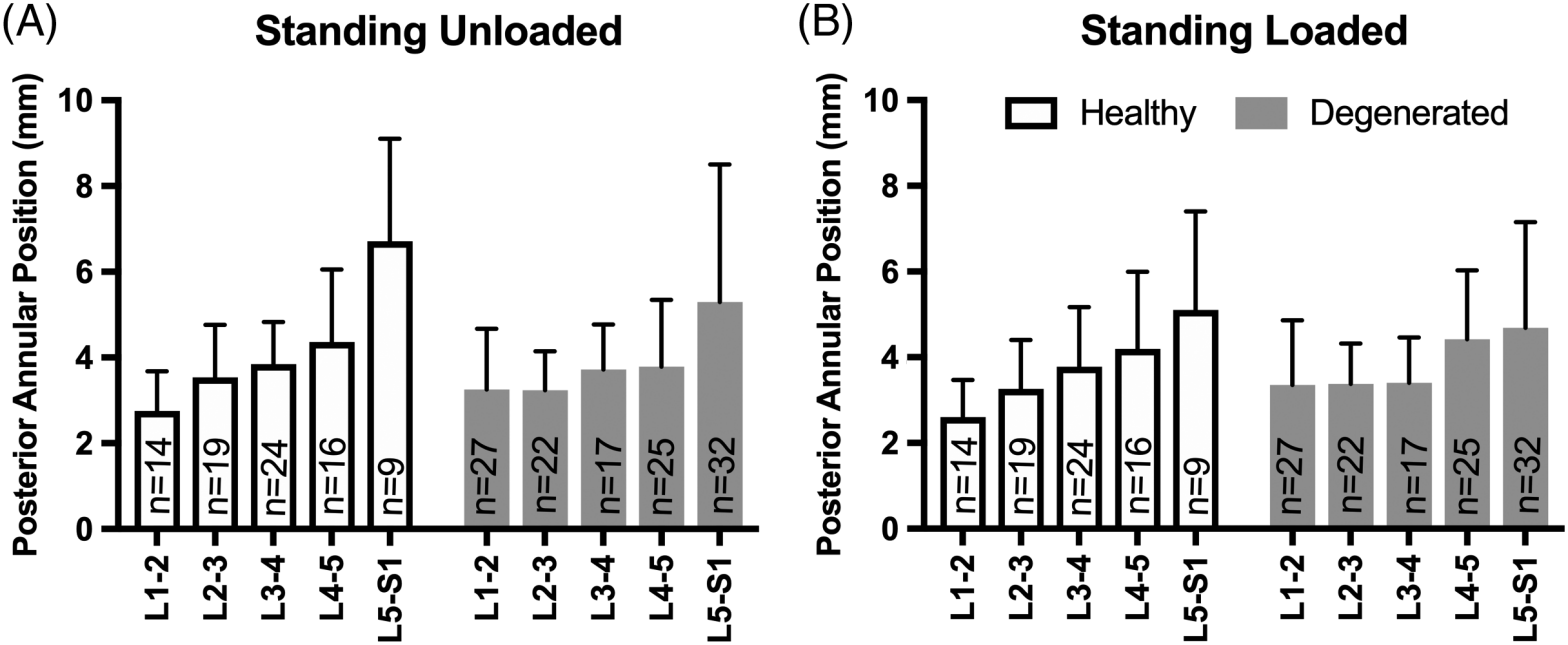
Intervertebral disc (IVD) degeneration at each level on posterior annular position in the standing unloaded and loaded conditions. A, The effect of IVD degeneration at each level on posterior annular position in degenerated IVDs is seen in the standing unloaded condition as compared with non‐degenerated IVDs. B, The effect of IVD degeneration at each level on posterior annular position in degenerated IVDs is seen in the standing loaded conditions as compared with non‐degenerated IVDs. There was no significant effect of IVD degeneration at any level amongst all participants standing with or without axial load (*P* > .205)

There were no differences in posterior annular position with loading between healthy and degenerated discs (Pfirrmann ≥ grade III) at any lumbar level (Figure 2A,B; *P* > .205).

### 3.3 Effect of axial load on local lordosis and disc heights

Changes in sagittal intervertebral angle were not associated with changes in posterior annular position between baseline and loaded conditions at any lumbar level (*r* < 0.267; *P* = .091‐.746). Intervertebral angles were significantly larger in the lower regions of the spine (*P* < .001), indicating increased local lordosis when moving in the caudal direction. There was also a trend for the main effect of load on intervertebral angles, in that angles were larger (more lordotic) at baseline (7.15° ± 1.63) than with load (6.77° ± 1.85, *P* = .064).

There was a main effect of level on disc height, in that disc height at the L5/S1 level was significantly smaller (6.3 mm, mean difference = 1.20) than all other levels (*P* < .001). There was a trend for baseline posterior disc heights to be larger at baseline (7.43 mm ± 1.46) than after loading (7.18 ± 1.57, *P* = .071). Additionally, there was a significant interaction (*P* = .006) between axial load and posterior disc height across levels, such that while the L1/2 disc exhibited an increase in disc height with loading, all other levels exhibited a decrease in disc height. However, of these differences, only the L3/4 and L4/5 discs were statistically significant (*P* < .022).

### 3.4 Effect of axial load on posterior annular position

**Figure 3:**
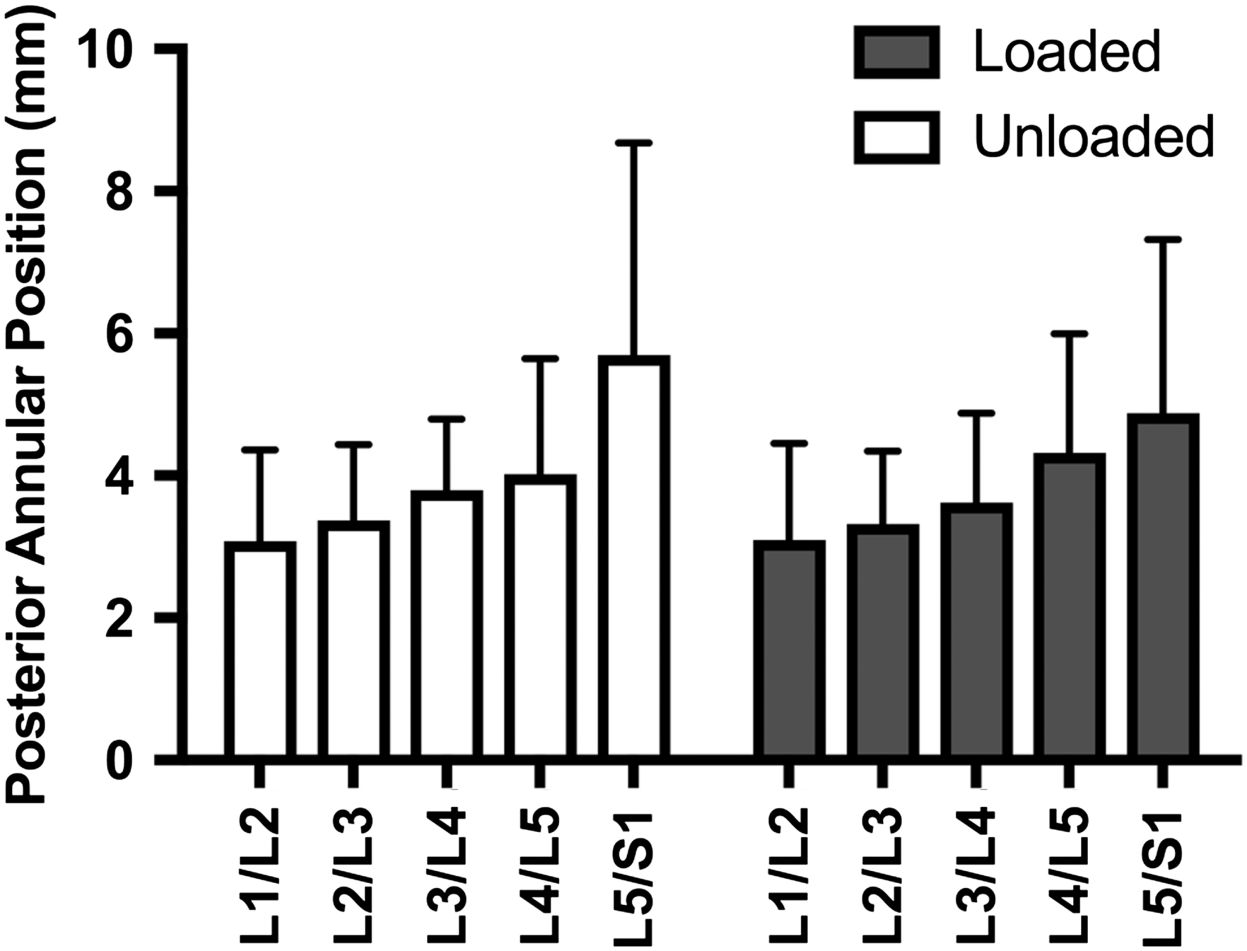
Intervertebral disc (IVD) measurements across lumbar levels in 41 active duty Marines. The Marines' posterior annular position was recorded in the standing unloaded (white) and standing loaded (gray) conditions at all levels. There was a significant main effect for IVD level (*P* < .035), but no effect of loading (*P* = .363)

Posterior annular position at baseline was found to be different across lumbar levels (Figure 3), with the L5/S1 level demonstrating greater values (more posterior protrusion beyond the vertebral border) than L1/L2 (mean difference = 2.63 mm), L2/3 (mean difference = 2.34 mm), L3/L4 (mean difference = 1.87 mm), and L4/5 (mean difference (1.69 mm), *P* < .035. There was no main effect of loading on posterior annular position (mean difference 0.16 mm reduction, *P* = .363). There was a trend for an interaction between loading and level, with the increase in posterior annular position from L4/5 to L5/S1 losing significance in the loading condition (*P* = .919), indicating that posterior annular position changes throughout the spine are reduced with loading at the L5/S1 level (Figure 3B).

### 3.5 Effect of larger baseline posterior annular positions on posterior annular position with load

**Figure 4:**
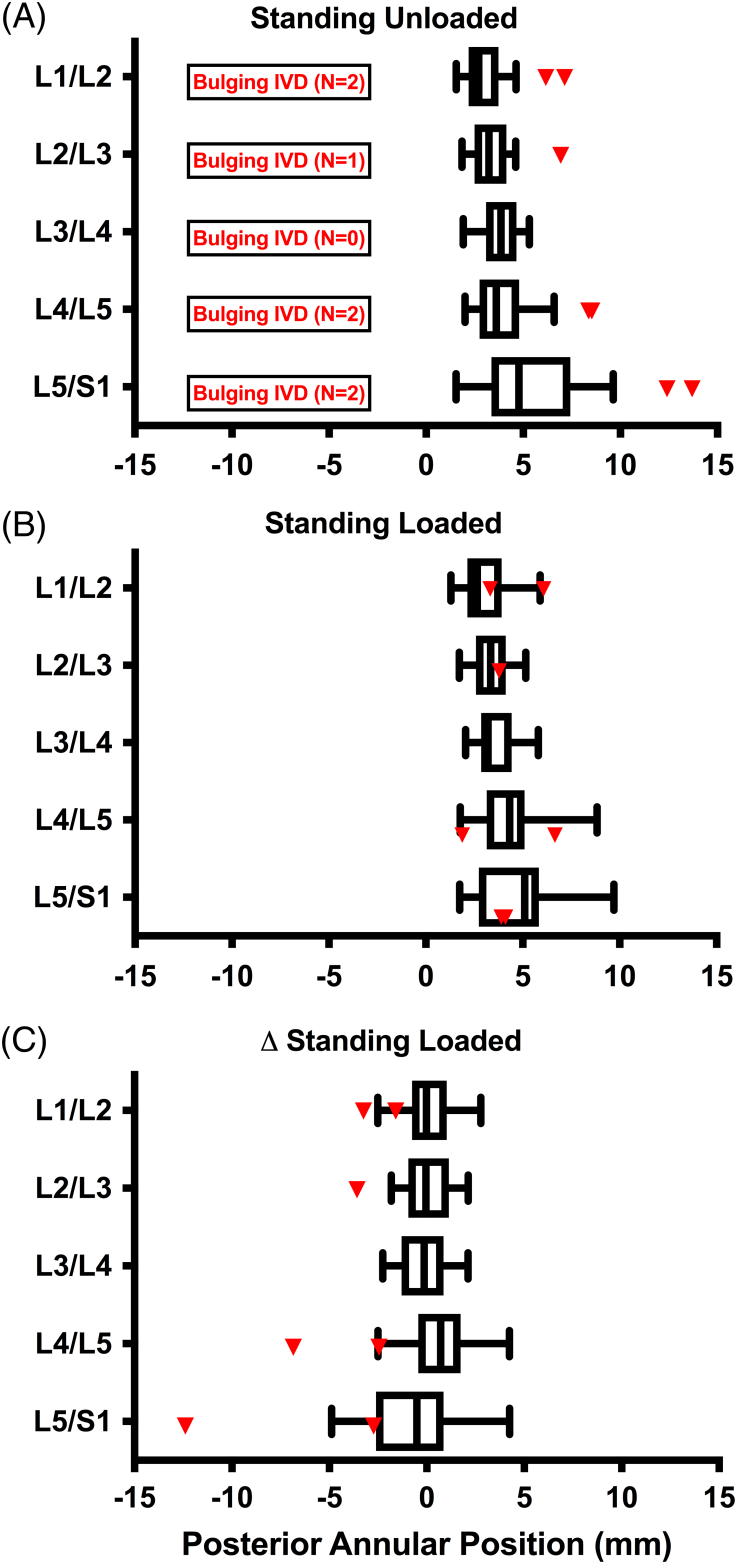
Intervertebral disc (IVD) behavior standing at baseline and with axial load. A, Posterior annular position of all participants at all lumbar levels when standing unloaded. Individuals whose IVD posterior annular positions were greater than the 95th percentile while standing unloaded were characterized as bulging IVDs (shown in red). B, Posterior annular position standing with load at all lumbar levels in normal (white) and bulging IVDs (red). C, Difference between posterior annular position when standing unloaded and standing with load at all lumbar levels in normal (white) and bulging IVDs (red)

**Figure 5:**
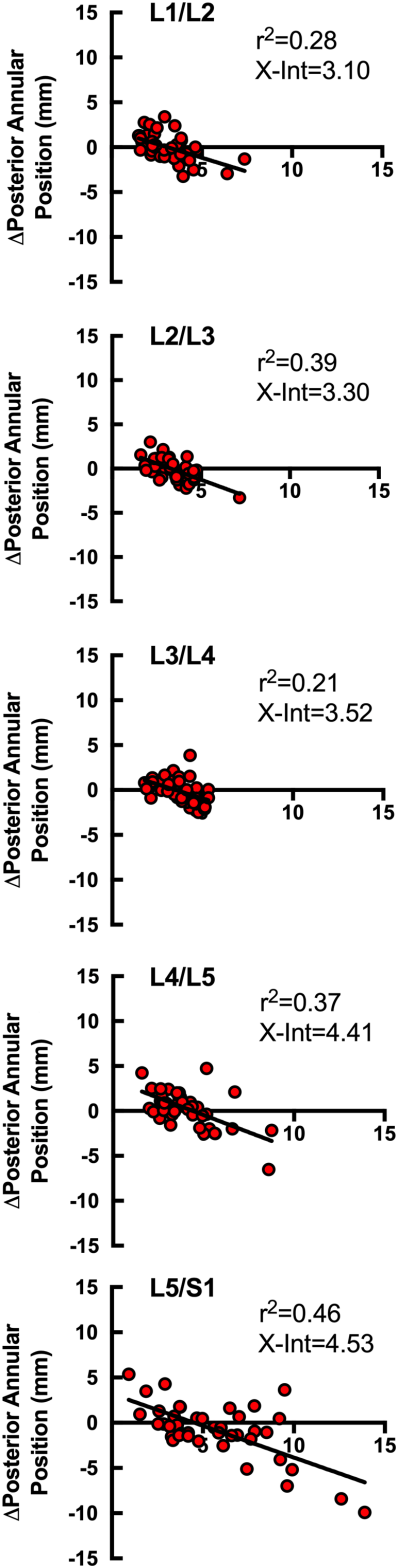
Relationship between posterior annular position when standing unloaded, and the difference in posterior annular position between standing unloaded and standing loaded positions. From top (L1/L2) to bottom (L5/S1), all lumbar levels are shown in all individuals under study with *r* ^2^ values. At all levels, there was a significant negative relationship between larger posterior annular position and a reduction in posterior annular position standing with axial load (*P* < .001). X‐Int, x‐intercept

In the standing unloaded condition, 15% of participants (6/41) showed evidence of baseline disc bulge at one or more level (7/205 IVDs), defined as posterior annular position greater than two SDs above the mean (Figure 4A). Qualitatively, those individuals with bulging discs at baseline demonstrated a greater reduction in posterior annular position (decrease in disc bulge) with load (Figure 4B,C). Quantitatively, there was a significant negative relationship between the baseline posterior annular position and the change in posterior annular position when loaded at all lumbar levels (*r* = −0.463 to −0.677, *P* < .002; Figure 5), with the L5/S1 level demonstrating the strongest relationship (*r* = −0.677; *P* < .001; Figure 5).

Generally, the point of inflection (x‐intercept) where IVD bulge began to reduce with axial load began at approximately 3 mm posterior in the midsagittal plane. IVDs with posterior annular positions less than 3 mm tended to exhibit increased magnitude of posterior annular position under axial load, whereas IVDs with posterior annular positions greater than 3 mm tended to display increasing posterior annular position reduction under axial load (Figure 5). This point of inflection was observed to increase at lower lumbar levels.

## 4 DISCUSSION

This study demonstrated that a large proportion of healthy active duty Marines showed signs of disc degeneration in the lumbar spine. However, these features did not affect changes in IVD kinematics (posterior annular position or sagittal intervertebral angle) with operationally relevant loads. Decreases in local lordosis with loading were most prominent at the lower lumbar levels (L4‐S1), but these decreases were not associated with changes in posterior annular position. Interestingly, we observed reductions in the magnitude of posterior annular position in the sagittal plane with load, which was most evident in discs that had larger posterior annular positions, or were bulging at baseline.

As IVDs degenerate, composition changes occur, such as hydration and height loss, potentially changing kinematic behavior in the final stages of degeneration in a way that alters spinal postural mobility.^11^ Examining bone marrow and vertebral endplate changes in symptomatic patients with degenerative disc disease, Hiyashi et al found that segments with increasingly pathologic endplates (higher Modic changes) had increased Pfirrmann grade and decreased angular motion leading to relative linearization of the lumbar spine.^21^ Previously, we have shown Marines with degeneration at the L5/S1 IVD demonstrated larger sacral postural alterations in response to axial load, as well as a reduced lumbar lordosis.^4^, ^9^ Prior literature also suggests that the most angular lordosis comes from the L4/L5 and L5/S1 vertebral segments,^22^ so the present study's findings support the concept that IVD kinematic changes occur at the most mobile segments of the lumbar spine, which are thought to be the most inferior levels. A similar investigation of IVD kinematic parameters was performed in individuals with diagnosed IVD pathology by Zou et al, who found that not only did discs with more degeneration tend to have larger bulges, but that these discs migrated posteriorly during extension without anterior migration in flexion at lower lumbar levels.^6^ Although this trend aligns with normal IVD kinematic behavior in response to movement,^23^ the findings by Hayashi et al and our results in individuals without clinically diagnosed disc pathology do not coincide with these findings. Hayashi et al found that superior lumbar segments with more degenerative changes (higher Modic changes) exhibited increasing disc bulge and decreasing angular motion, but this was not the case at L5/S1.^21^ While our population under axial loading conditions demonstrated no relationship between IVD degeneration and posterior disc protrusion, our results similarly demonstrated that disc bulge was not found to increase at lower lumbar segments. Contrarily, the addition of axial load appeared to cause reduction in posterior annular protrusion, indicating decreasing disc bulge at lower lumbar levels.

While our data support previous literature demonstrating lumbar spine postural adaptation (ie, decreased lordosis) rather than IVD compression (ie, decreased disc height) in response to axial load,^24^ it is unclear how these loads result in reductions in posterior annular position. One possible mechanism is that the posterior longitudinal ligament (PLL) may become stretched due to posterior IVD distraction in response to angular changes, causing the IVD to shift anteriorly as local lordosis is reduced. Given the participants were not instructed to maintain any particular position for bearing the axial loads during image acquisition, there could be different recruitment of paraspinal musculature (iliocostalis, longissimus, spinalis, and multifidus) that may affect global posture and IVD kinematics. However, we found no significant relationship between the change in IVD angle and change in posterior annular position with load, which suggests a different mechanism.

Rodriguez‐Soto et al demonstrated linearization of the inferior lumbar spine with increased posterior disc heights in response to posterior axial load. While our results did not show changes in posterior disc heights or intervertebral angles with less axial load than was used in their study, we too were able to observe changes in the inferior lumbar spine. In the present study, the increased posterior annular position with loading most obvious at L4/L5 and L5/S1 may also be related to what Rodriguez‐Soto et al postulated to be an effort to relocate center of mass. If increased posterior annular position is sequelae of alterations to center of mass with load carriage, and these alterations risk premature degeneration of the disc, patients may benefit from restoration of native spinal posture. Clinical prevention and treatment should therefore be aimed at restoring lordosis at baseline. This may be possible with interventions aimed at reducing axial loading, strengthening supporting musculature with physical therapy to redistribute the axial load, or with load redistributing devices (ie, backpacks with hip harnesses or internal frames). However, such interventions may be impractical in the population under study, given the requirements of their occupations. Although our imaging findings have demonstrated alterations to lumbar IVD kinematics (posterior annular position and local lordosis) in response to light axial load in Marines without clinically diagnosed spinal pathology, there was no clear association with degeneration. Therefore, imaging findings alone may not be sufficient to guide clinical practice in patients, so clinical exam must correlate with imaging findings to best advise patients how to preserve the health of their spine.

In this study, we limited our analysis of disc bulge to the mid‐sagittal plane. The prevalence of central versus paracentral disc bulge in healthy populations is unclear. In symptomatic cases, if a disc is protruded or extruded, then paracentral location is most commonly observed.^25^, ^26^ However, even when a disc bulge is paracentral, it is most often diffuse, and able to be seen to some extent in the mid‐sagittal plane.^26^ In asymptomatic individuals or in individuals with mild disc bulges—not protrusions or extrusions—the location of the bulge is more likely to be central.^26^ As diagnosed spinal pathology was an exclusion criterion for this study, and the lack of paracentral disc bulges was visually confirmed, the volunteers in this study are within the latter group.

There are three main limitations to the study. To acquire the imaging data for analysis, an elastic band was used to gently secure the coil to the volunteers' low back. While this may influence posture, the band and the coil are relatively light (approximately 1 kg), and the posture of patients did not appear to change when it was attached. Additionally, we were unable to resolve the PLL due to the short T2 relaxation of collagenous tissues. Development of new ultra‐short TE pulse sequences may provide insight into the health and function of the PLL under axial load. We used the posterior border of the annulus fibrosis (AF) as a proxy for disc bulge, which may not be the most accurate characterization of IVD movements. Rather, we may be observing abnormal nucleus pulposus migration with respect to adjacent vertebral bodies.^23^ Lastly, Marines are exposed to significant conditioning, as well as physical demand, which may contribute to differences in findings compared to age and sex matched civilians. Such characteristics may impact the external validity of our findings and limit their applicability to the general population. Future work should be directed toward localizing IVD migration in multiple planes to better characterize kinematic responses to axial load. The findings of this analysis warrant further investigation into axial loading and resultant IVD kinematic changes in hopes of elucidating its unique alterations to disc morphology in a highly active population.

## DISCLOSURE OF INTERESTS

K.O.: Nothing to disclose. D.B.B.: Nothing to disclose. B.S.: Nothing to disclose. K.K.: Nothing to disclose. S.R.W.: Nothing to disclose.

## AUTHOR CONTRIBUTIONS

D.B., B.S., collected data. K.O., D.B, K.K., and S.W. designed the research question. K.O., D.B., and B.S. processed the data. K.O. processed statistics and wrote the manuscript. All authors reviewed and approved the manuscript.
